# Efficacy of pregabalin in post-traumatic peripheral neuropathic pain: a randomized, double-blind, placebo-controlled phase 3 trial

**DOI:** 10.1007/s00415-018-9063-9

**Published:** 2018-09-21

**Authors:** John Markman, Malca Resnick, Scott Greenberg, Nathaniel Katz, Ruoyong Yang, Joseph Scavone, Ed Whalen, Gabriela Gregorian, Bruce Parsons, Lloyd Knapp

**Affiliations:** 1 Translational Pain Research Program, Department of Neurosurgery, University of Rochester Medical Center, 601 Elmwood Avenue, Box 670, Rochester, NY 14642 USA; 2 Pfizer Inc, New York, NY USA; 3 Analgesic Solutions, Natick, MA USA

**Keywords:** Neuropathic pain, Post-traumatic neuropathic pain, Post-surgical neuropathic pain, Pregabalin

## Abstract

The growing need for symptomatic treatment of post-traumatic neuropathic pain (PTNP) continues to be unmet. Studies evaluating the efficacy of pregabalin for reducing neuropathic pain following trauma and surgery yielded positive results over ≤ 8-week treatment. To assess the efficacy and tolerability of pregabalin over 3 months in patients with PTNP, a randomized, double-blind, placebo-controlled, parallel-group trial evaluated patients with PTNP at 101 centers in 11 countries—the longest, largest such trial. Adults diagnosed with PTNP were randomly assigned (1:1) to 15 weeks of pregabalin (flexibly dosed 150–600 mg/day) or matching placebo. Primary efficacy analysis was by mixed-model repeated measures comparing change from baseline to week 15 in weekly mean pain scores between active and placebo groups. Evaluable patients included 274 in the pregabalin group and 265 in the placebo group. Trauma was surgical in 49.6% of patients, non-surgical in the remainder. The primary efficacy analysis showed no statistically significant difference between pregabalin and placebo groups in the change from baseline to week 15 [mean difference, − 0.22 points (95% confidence interval, 0.54–0.10); *p* = 0.1823]. However, comparisons for key secondary outcome measures yielded *p* values < 0.05 favoring pregabalin. Consistent with the known safety profile of pregabalin, the most common adverse events were dizziness and somnolence (14.6 and 9.9% of patients, respectively) with pregabalin (vs 4.2 and 3.4% with placebo). These findings demonstrate the feasibility of conducting a large, phase 3 registration trial in the heterogeneous PTNP study population.

*ClinicalTrials.gov* NCT01701362.

**Electronic supplementary material:**

The online version of this article (10.1007/s00415-018-9063-9) contains supplementary material, which is available to authorized users.

## Introduction

Diverse types of nerve injury are recognized as triggers of chronic post-traumatic neuropathic pain (PTNP), including post-surgical syndromes [1–3]. Tissue injury may chronically alter peripheral nociceptive processing, shifting pain from acute to chronic [4, 5]. The need for symptomatic treatment of PTNP is increasing but remains inadequately addressed [6–8].

Pregabalin, an alpha_2_-delta (α_2_δ) ligand (gabapentinoid), is approved in the United States for the treatment of three neuropathic pain (NeP) conditions: diabetic peripheral neuropathy (DPN), post-herpetic neuralgia (PHN), and post-spinal cord injury (SCI) [9]. An 8-week randomized clinical trial demonstrated the efficacy of pregabalin for the management of chronic post-traumatic/post-surgical pain [10].

A study of longer duration was designed to meet the US regulatory standard for a chronic pain indication: 12 weeks of maintenance or fixed dosing [11]. Methodologic features were incorporated to increase assay sensitivity for the detection of an analgesic signal in this heterogeneous patient population [12]. The primary objective was to compare the efficacy of pregabalin (flexibly dosed, 150–600 mg/day) versus placebo in the treatment of PTNP. Secondary objectives compared the efficacy of pregabalin vs placebo with respect to overall status, pain-related activity limitation, and sleep, in addition to safety and tolerability assessments.

## Methods

### Study patients

Eligible patients were aged ≥ 18 years and had PTNP for ≥ 6 months after a surgical or non-surgical traumatic event (e.g., history of a motor vehicle accident, fall, sports injury, knee or hip replacement, hernia repair, thoracotomy, mastectomy, focal/localized burns, or crush injury), a mean score of ≥ 4 in pain recall for the past week at screening, and a mean score of ≥ 4 and ≤ 9 on a 0–10 numeric rating scale (NRS) of average pain (0, “no pain” to 10, “worst possible pain”) based on ≥ 4 daily diary scores from the last week of a single-blind baseline screening period (5–14 days before randomization). Peripheral nerve(s) implicated in the pain was identified to confirm nerve trauma, and pain was categorized as neuropathic based on prespecified criteria (i.e., neurologic exam, study-specific PTNP assessment including use of the PainDETECT questionnaire to identify neuropathic components of back pain [13]) and diagnostic tests (e.g., electromyography, nerve conduction tests, skin or nerve biopsy) if available. While PainDETECT was a screening assessment and used as part of the initial diagnostic assessment, since it is not specifically validated for this indication, this instrument itself did not determine eligibility in the study.

Each neuropathic symptom or sign, mapped separately, was submitted to a team of independent neurologists (contracted by Analgesic Solutions, Natick, MA, USA) who determined the plausibility of matching a PTNP syndrome with respect to the history, anatomic distribution of reported pain, and associated signs identified on neurologic examination in the corresponding body region.

Exclusion criteria included NeP due to PHN, DPN, complex regional pain syndrome, and other conditions; other sources of pain that might confound assessment of PTNP; disallowed concomitant medications; nonpharmacologic treatments for PTNP; severe or acute medical or psychiatric conditions; or clinically significant laboratory abnormalities. Patients scoring ≥ 15 on the Patient Health Questionnaire (PHQ-8) at screening or who were at risk based on Columbia-Suicide Severity Rating (C-SSRS) responses were recommended for evaluation by a mental health professional prior to randomization [14–16]. Prohibited medications included opioids, local anesthetics, topical and intraspinal steroids, antiepileptics, and antipsychotics. Allowed medications included stable regimens of nonsteroidal anti-inflammatory drugs (NSAIDs), non-opioid analgesics, antidepressants [including serotonin-specific reuptake inhibitors (SSRIs), tricyclic antidepressants, and serotonin–norepinephrine reuptake inhibitors (SNRIs)], tramadol and triptans, and/or sleep medications; acetaminophen ≤ 3 g/day was allowed as rescue medication.

### Study design and procedures

Eligible patients were randomized at 101 centers in 11 countries (Bulgaria, Canada, Denmark, Germany, Hungary, Poland, Romania, Sweden, South Africa, South Korea, and the United States). Following a single-blind screening period, the 15-week double-blind treatment period comprised 3 weeks of dose titration/optimization and 12 weeks of maintenance treatment (Supplementary Fig. S1). After randomization, clinic visits occurred every 3 weeks.

Patients were randomized (1:1) to pregabalin or matching placebo. The pregabalin dose was individually optimized via telephone contact to 150, 300, 450, or 600 mg/day over the titration period, with 4 days at each dose before titration to the next level. Dose adjustments were not allowed during the maintenance period, except for a single dose reduction if the investigator judged it necessary for tolerability.

### Outcome measures

The primary outcome was pain rated in a diary completed by telephone each evening between 7 pm and midnight. Patients were asked to select the number that best described their NeP during the past 24 h on an 11-point NRS ranging from 0 (“no pain”) to 10 (“worst possible pain”). During this call, patients were also asked to select the number that best described how NeP had interfered with their sleep during the past 24 h, on an NRS from 0 (“pain does not interfere with sleep”) to 10 (“pain completely interferes with sleep [unable to sleep due to pain]”).

Secondary outcome measures at randomization and endpoint included the patient-reported Medical Outcomes Study-Sleep Scale (MOS-SS) [17], Brief Pain Inventory-Short Form (BPI-sf) [18], European Quality of Life 5-Dimensions Questionnaire (EQ-5D) [19], Healthcare Utilization Economic Assessment, and Work Productivity and Activity Impairment Questionnaire-Specific Health Problem (WPAI-SHP) [20]. Patients completed the Patient Global Impression of Change (PGIC) using a 7-point scale from “very much worse” to “very much improved”. At endpoint, only the BPI-sf was summarized for the pain severity index score (average of 4 individual pain scores) and pain interference index score (average of 7 individual interference scores).

### Statistical analyses

Sample size was calculated using estimates of variance and treatment difference from a previous PTNP trial. A sample of 235 patients per arm would provide 90% power to detect a treatment difference from placebo of 0.6 with respect to change from baseline to week 15 in mean pain scores (MPS), assuming standard deviation (SD) of 2.0 and type I error rate of 0.05. A preplanned, unblinded interim analysis to re-estimate the sample size up to a maximum of 700 patients was performed by an independent data and safety monitoring board when approximately 80% of 470 patients had completed or discontinued from the study. The interim analysis was not intended to stop the study early for any efficacy claim.

The prespecified primary analysis compared change from baseline to week 15 in the MPS. The primary analysis employed mixed-model repeated measures (MMRM), using SAS PROC MIXED with model terms of treatment, trauma type, country, week, treatment-by-week interaction, and baseline MPS as covariates. Week was used as a class variable. A multiple imputation (MI) method was used to impute missing MPS for the primary efficacy analysis, with poor outcomes imputed for withdrawals due to adverse events (AEs) or lack of efficacy.

Secondary analyses compared the change from baseline in weekly MPS and weekly mean sleep interference score with an MMRM model using model terms of treatment, trauma type, center, week, treatment-by-week interaction, and baseline MPS as covariates, without use of an MI algorithm. For sensitivity analyses, analysis of covariance (ANCOVA) with model terms of treatment, center, trauma type, and baseline value analysis was applied to the change from baseline to week 15 in MPS with imputation methods of baseline observation carried forward (BOCF), last observation carried forward (LOCF), and modified baseline observation carried forward (mBOCF), which applied the BOCF rule for patients discontinued due to AEs and the LOCF rule for patients discontinued for any other reason. Other continuous secondary endpoints were also analyzed with this ANCOVA model.

Responder analyses compared the percentage of participants who achieved a 30% and 50% reduction in MPS from baseline to weeks 1–15 using a generalized linear model with a logistic link function. The model included categorical effects of treatment, center, trauma type, week, and treatment-by-week interaction, as well as a continuous baseline MPS. PGIC was analyzed at the endpoint using the Cochran–Mantel–Haenszel (CMH) test stratified for center and trauma type.

A *p* value < 0.05 was considered statistically significant for all analyses. Adjustments for multiple secondary analyses were not made. The intention-to-treat (ITT) population, defined as all randomized patients who received at least one dose of study drug, was used for all analyses. A per-protocol population was defined as a subset of the ITT population who completed the study and did not have major protocol deviations with the potential to affect the primary efficacy analysis. The per-protocol population was used only in a sensitivity analysis of the primary efficacy endpoint.

## Results

### Demographic and baseline characteristics

**Fig. 1:**
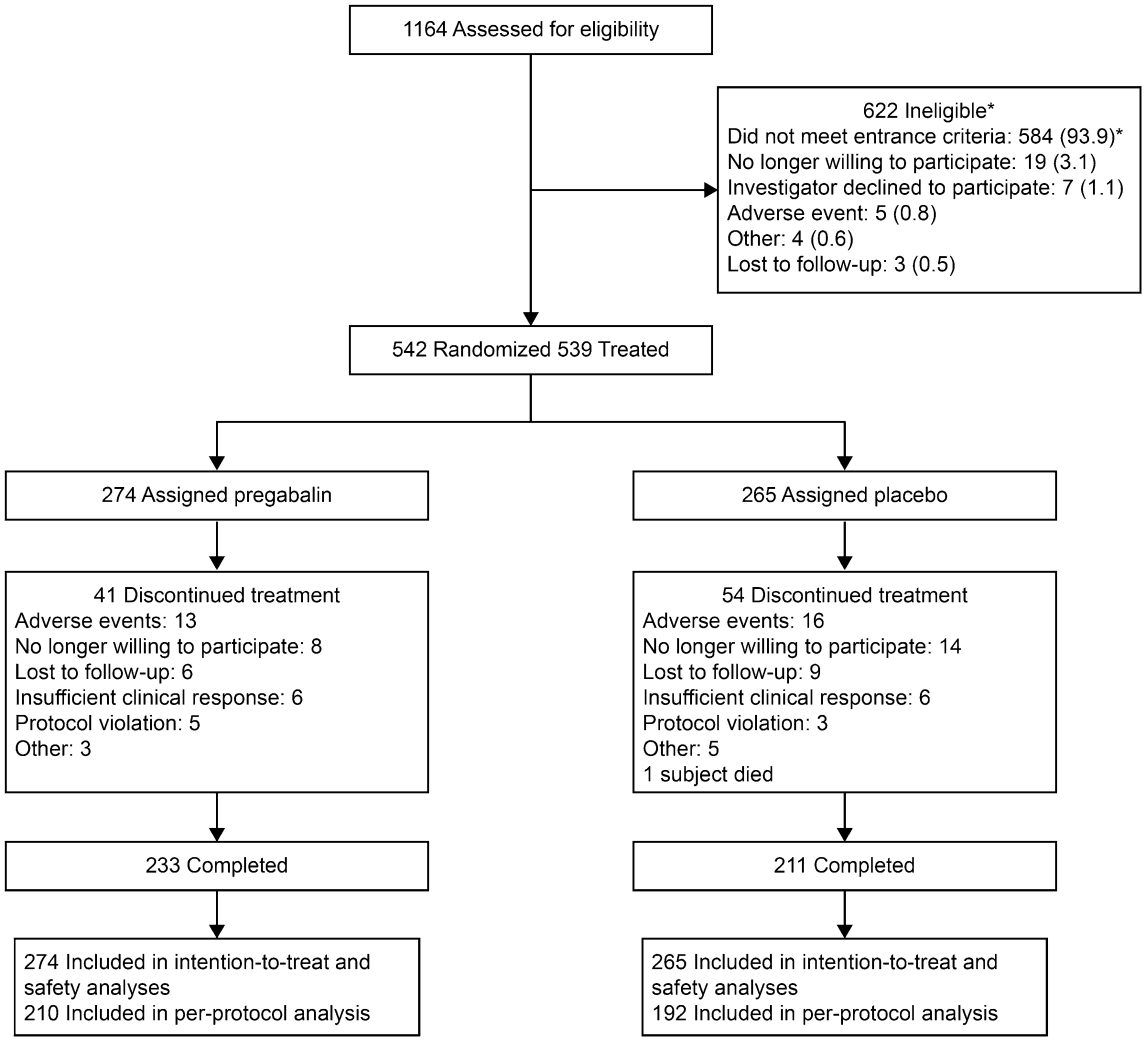
Trial profile. Subjects may have met more than one criterion for exclusion. Of 584 not meeting eligibility criteria, 190 did not meet requirements for neuropathic pain assessment; 71 did not meet the required duration of PTNP; 69 did not meet pain diary criteria prior to randomization; 65 were unwilling/unable to comply with study procedures; 44 had exclusionary pain conditions; 43 did not have the implicated peripheral nerve identified; 34 had other exclusionary NeP conditions; 34 had creatinine clearance ≤ 60 mL/min; and 22 were taking prohibited medications

This study was conducted between 31 October 2012 and 4 August 2015. Of 1164 patients screened for inclusion, 622 did not meet the inclusion criteria, most often because of failure to meet eligibility criteria (*n* = 584; 93.9%), specifically, the NeP criteria (*n* = 190). A total of 542 patients were randomized, 3 of whom were not treated (pregabalin, *n* = 274; placebo, *n* = 265) (Fig. 1).

Approximately half the patients were male; mean age was 53 years (range, 20–85 years). Most patients were white (79.7%). Approximately half the patients had pain from surgery, and the remainder had pain from other traumatic injuries. All randomized patients had a primary diagnosis of peripheral nerve injury, with the nerves most commonly affected (i.e., amounting to > 5% of cases) as follows: peroneal (10.1%), ulnar (6.6%), sural (6.1%), median (6.0%), sciatic (5.9%), radial (5.6%), lateral cutaneous nerve of the thigh (5.3%), and other (6.3%). Demographic characteristics were similar between treatment groups. Mean duration between symptom onset and enrollment in the trial was 8.0 years in both treatment groups. The MPS from the baseline week of the daily NRS diary was 6.41 in the pregabalin group and 6.54 in the placebo group (Table 1).

**Table 1:** Baseline characteristics (randomized population)

Demographic characteristics	Pregabalin (*n* = 275)	Placebo (*n* = 267)	Total (*N* = 542)
Age (years)			
Mean (SD)	52.8 (12.9)	53.5 (12.6)	53.1 (12.8)
Range	20–81	20–85	20–85
Age, years, no. (%) of patients			
18–44	60 (21.8)	61 (22.8)	121 (22.3)
45–64	163 (59.3)	156 (58.4)	319 (58.9)
≥ 65	52 (18.9)	50 (18.7)	102 (18.8)
Race, no. (%) of patients			
White	217 (78.9)	215 (80.5)	432 (79.7)
Black	47 (17.1)	46 (17.2)	93 (17.2)
Asian	7 (2.5)	3 (1.1)	10 (1.8)
Other	4 (1.5)	3 (1.1)	7 (1.3)
Weight (kg)			
Mean (SD)	85.9 (20.0)	86.2 (19.0)	86.1 (19.5)
Range	49.1–193.7	45.4–166.0	45.4–193.7
Height (cm)			
Mean (SD)	170.1 (10.1)	168.6 (9.4)	169.4 (9.8)
Range	142.2–198·0	140.0–198.1	140.0–198.1
Trauma type, no. (%) of patients			
Surgical	131 (47.6)	138 (51.7)	269 (49.6)
Non-surgical	144 (52.4)	129 (48.3)	273 (50.4)
Baseline mean pain (daily NRS)			
Mean (SD)	6.41 (1.3)	6.5 (1.3)	
Questionnaire: PainDETECT [13]	*N* = 274	*N* = 265	
Mean (SD)	23.1 (5.52)	22.6 (5.52)	
Range	2–36	3–38	

*NRS* numeric rating scale, *SD* standard deviation

Prior exposure to gabapentinoids (40 patients in total) was higher in the placebo group (9.4%) than in the pregabalin group (5.5%). Concomitant pain drug treatments (continuing stable treatment at randomization) were taken by 120 (43.8%) patients in the pregabalin group and 130 (49.1%) patients in the placebo group. The most common concomitant pain drugs were ibuprofen, acetaminophen, and tramadol. The proportions of patients taking antidepressants for any reason were similar between the treatment groups, comprising less than one-tenth of patients in either group, with a smaller subset of patients taking tricyclic antidepressants or serotonin–norepinephrine reuptake inhibitors. Rescue drug treatments were taken by 16 patients in the pregabalin group and by 37 patients in the placebo group.

At the end of the dose optimization period, the mean maintenance dose for evaluable pregabalin-treated patients (*n* = 256) was 473.7 mg/day. The maintenance dose of pregabalin was 600 mg/day for 150 (58.6%) patients, 450 mg/day for 54 (21.1%) patients, 300 mg/day for 28 (10.9%) patients, and 150 mg/day for 24 (9.4%) patients.

### Efficacy

#### Primary analysis

The primary analysis did not demonstrate a significant difference between groups in the mean change of pain scores from baseline to week 15 [pregabalin vs placebo, − 0.22; 95% confidence interval (CI) − 0.54 to 0.10; *p* = 0.1823]. At week 15, pain scores in both groups had improved compared with baseline [least-squares (LS) mean change from baseline: pregabalin, − 2.12; placebo, − 1.90]. The results were similar when missing data were imputed using sensitivity analyses LOCF, BOCF, and mBOCF.

**Fig. 2:**
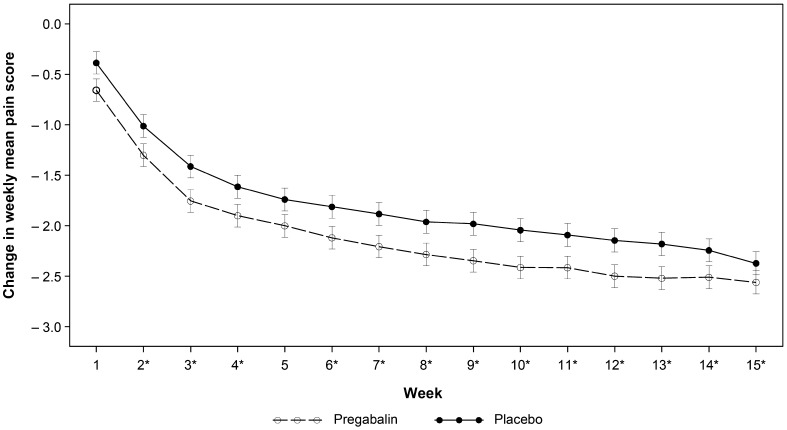
Change from baseline in weekly mean pain score (daily pain NRS; ITT population). *Unadjusted *p* < 0.05 from MMRM analysis. Changes in weekly mean pain score ± standard error were estimated from mixed-model repeated measures (MMRM) model. Weekly mean pain numerical rating scale (NRS) scores are derived from the daily pain NRS and calculated as the mean of the available scores in the 7 days. Generally, week “n” mean pain score is defined as the mean of the 7 daily diary pain ratings from day 2 + 7*(*n*–1) to day 1 + 7*n. At least four entries within the last 7 days are required to calculate a mean score. NRS ranged from 0 (“no pain”) to 10 (“worst possible pain”), with higher scores indicating increased pain. ITT intention to treat

*Weekly assessments* At all weeks, the pregabalin-treated group had greater improvement in weekly MPS than the placebo-treated group, although a relative increase in placebo response during the final 2 weeks, 14 and 15, was noted. Differences were statistically significant (*p* < 0.05) from week 2 to 13, except for week 5 (Fig. 2). In addition, the overall mean was also statistically significant (*p* < 0.05), with a treatment difference of − 0.31.

The 30 and 50% responder status was defined for each patient based on the percentage change in the MPS from baseline to each week, 1–15. Overall, there were more pregabalin 30% and 50% pain responders when compared with the placebo group. The incidence of responders generally increased over the first 3 weeks of treatment for both 30 and 50% responders. Responder data for weeks 1–3, 14, and 15 are summarized for brevity (responder percentage [week, number responders/number observed at week, *p* value pregabalin vs placebo]). Pregabalin 30% responders were 11.9% (week 1, 31/260; *p* = 0.0028), 27.2% (week 2, 69/254; *p* = 0.0360), 38.9% (week 3, 98/252; *p* = 0.0235), 57.4% (week 14, 128/223; *p* = 0.4245), and 57.7% (week 15, 113/196; *p* = 0.8464). Placebo 30% responders were 5.0% (week 1, 13/258), 20.1% (week 2, 49/244), 30.2% (week 3, 74/245), 54.3% (week 14, 113/208), and 58.3% (week 15, 109/187). Pregabalin 50% responders were 4.6% (week 1, 12/260; *p* = 0.1633), 11.4% (week 2, 29/254; *p* = 0.0652), 22.6% (week 3, 57/252; *p* = 0.0039), 37.7% (week 14, 84/223; *p* = 0.0314), and 39.8% (week 15, 78/196; *p* = 0.1889). Placebo 50% responders were 2.3% (week 1, 6/258), 7.0% (week 2, 17/244), 13.5% (week 3, 33/245), 29.8% (week 14, 62/208), and 34.2% (week 15, 64/187). Differences were statistically significant for pregabalin compared with placebo (*p* < 0.05, generalized linear model) for 30% responders at weeks 1–3, and for 50% responders at weeks 3–5, 7, 9, and 11–14. In the BPI-sf mean pain severity index, a statistically significant change from baseline to week 15 favored pregabalin over placebo (*p* = 0.0050; ANCOVA). The LS mean (standard error) change was − 2.40 (0.13) in the pregabalin group and − 1.95 (0.13) in the placebo group. In the mean pain interference index, a statistically significant change from baseline also favored pregabalin over placebo (*p* = 0.0168) (Table 2).

**Table 2:** Summary of efficacy results: ITT population

Outcome measure	Screening/baseline (s.d.)		LS mean change from baseline (s.e.) [95% CI]		LS mean difference (s.e.) [95% CI]	*p* value
	Pregabalin (*N* = 274)	Placebo (*N* = 265)	Pregabalin	Placebo		
Primary efficacy endpoint, week 15 mean pain	6.41 (1.29)	6.54 (1.31)	− 2.12 (0.15) [− 2.42, − 1.82]	− 1.90 (0.16) [– 2.21, − 1.60]	– 0.22 (0.16) [– 0.54, 0.10]	0.1823
Week 1–15 overall mean pain effect^a^	6.41 (1.29)	6.54 (1.31)	– 2.10 (0.10) [– 2.29, − 1.90]	− 1.79 (0.10) [–1.99, − 1.60]	− 0.31 (0.12) [– 0.55, − 0.07]	0.0117
Sleep interference, week 15	4.97 (2.30)	4.99 (2.27)	− 2.29 (0.11) [− 2.51, − 2.07]	− 1.86 (0.11) [− 2.08, − 1.63]	− 0.43 (0.15) [− 0.71, − 0.14]	0.0031
Endpoint BPI pain severity	5.95 (1.50)	5.90 (1.50)	− 2.40 (0.13) [− 2.66, − 2.15]	− 1.95 (0.13) [− 2.21, − 1.69]	− 0.46 (0.16) [− 0.77, − 0.14]	0.0050
Endpoint BPI pain interference	4.07 (2.16)	4.06 (2.11)	− 1.72 (0.13) − 1.97, − 1.46]	− 1.33 (0.13) [− 1.59, − 1.07]	− 0.38 (0.16) [− 0.70, − 0.07]	0.0168

*BPI* brief pain inventory, *CI* confidence interval, *ITT* intention to treat, *LS* least squares, *SD* standard deviation, *SE* standard error

Primary analyses and sleep: mixed-model repeated measures, intention-to-treat population; Brief Pain Inventory-Short Form analysis of covariance

^a^Overall is for each subject to pool pain score for all post-baseline weeks

At week 15, the PGIC ratings of improvement were statistically significantly higher in the pregabalin group than the placebo group. More patients in the pregabalin group (*n* = 157; 60.9%) than the placebo group (*n* = 120; 48.2%) reported that they were very much or much improved on the PGIC (*p* = 0.0029, CMH). Two additional predefined approaches for PGIC analysis, involving different groupings of the categories of improvement (“very much” or “much improved” vs all other groups; and categories comprising “any improvement”, “no change”, “any worsening”), also demonstrated statistically significant results favoring pregabalin.

*Sleep* The weekly mean sleep interference score at week 15 showed significantly greater improvement in the pregabalin group than in the placebo group [difference in adjusted mean change (pregabalin vs placebo) − 0.43; 95% CI, − 0.71 to − 0.14; *p* = 0.0031]. Adjusted mean change in sleep interference from baseline to week 15 in the pregabalin and placebo groups was − 2.29 (0.11) and − 1.86 (− 0.11), respectively. Statistically significant differences favoring pregabalin were observed at all weeks (Supplementary Fig. S2).

#### Safety

Pregabalin was well tolerated in this study, and the safety profile was consistent with or demonstrated fewer AEs when compared to the known profile of pregabalin in other NeP conditions [9]. Adverse events, most frequently dizziness and somnolence, occurred more frequently in the pregabalin group than the placebo group (Table 3). All pregabalin dose levels used in the study were combined for reporting AEs consistent with a flexible dose design in which doses were titrated based on patients’ responses and treatment tolerability. Overall, 138 (50.4%) patients in the pregabalin group and 106 (40.0%) in the placebo group experienced at least one AE. Two patients in the pregabalin group and seven in the placebo group experienced serious adverse events (SAEs), none of which was considered related to treatment. One death occurred in the placebo group. There were no clinically significant findings with respect to changes in laboratory values, vital signs, physical and neurologic examinations, or electrocardiogram.

**Table 3:** Adverse events (all-causality) experienced by ≥ 2% of patients in either treatment group by preferred term: safety analysis population^a^

No. (%) of patients with adverse events (treatment related) by preferred term	Pregabalin (*N* = 274) No. (%)	Placebo (*N* = 265) No. (%)
Dizziness	40 (14.6)	11 (4.2)
Somnolence	27 (9.9)	9 (3.4)
Fatigue	14 (5.1)	10 (3.8)
Nausea	14 (5.1)	8 (3.0)
Headache	12 (4.4)	8 (3.0)
Vertigo Back pain Disturbance in attention	12 (4.4) 8 (2.9) 8 (2.9)	1 (0.4) 5 (1.9) 0
Memory impairment Nasopharyngitis Constipation	7 (2.6) 7 (2.6) 6 (2.2)	0 8 (3.0) 4 (1.5)
Sedation Pain in extremity Insomnia	6 (2.2) 2 (0.7) 2 (0.7)	0 6 (2.3) 6 (2.3)

^a^Includes data up to 999 days after last dose of study drug. Medical Dictionary for Regulatory Activities **(**MedDRA) v18.1) coding dictionary applied

There were 53 (19.3%) and 16 (6.0%) patients in the pregabalin and placebo groups, respectively, who had dose reductions or temporary discontinuation of treatment due to AEs. Treatment-emergent (all-causality) AEs led to permanent discontinuation from the study for 13 (4.7%) patients in the pregabalin group and 15 (5.7%) patients in the placebo group. Dizziness led to permanent discontinuation of treatment for two patients in the pregabalin group and one in the placebo group. Discontinuation for somnolence occurred for two patients in the pregabalin group.

## Discussion

This randomized, double-blind, placebo-controlled, parallel-group study of patients with PTNP did not demonstrate a statistically significant treatment effect with pregabalin on mean pain intensity scores at the prespecified primary efficacy endpoint of the study, week 15. However, the primary efficacy parameter (change in mean pain intensity scores) was statistically significant at most other time points, and several secondary outcomes related to analgesic efficacy and quality of life improved with pregabalin compared with placebo treatment. Pregabalin was superior to placebo in reducing pain severity and interference with daily function as measured by the BPI-sf. A significantly greater number of patients in the pregabalin group described their overall status as very much improved or much improved when comparing their assessment on the PGIC during the last week of treatment (i.e., week 15) to that at study outset. Finally, there was a consistent and significant reduction in pain interference with sleep over the course of the study.

This was the first large phase 3, randomized, controlled trial designed to evaluate the analgesic efficacy of pregabalin in PTNP for registration purposes in the United States. The conduct of this trial indicates that a large, multinational, phase 3 trial of pregabalin in PTNP is feasible. The significant effect of pregabalin on a number of secondary endpoints, including second measure of pain intensity (BPI-sf) raises the possibility that the absence of a statistically significant difference on the primary endpoint may be partly attributed to additional factors individually or in aggregate including (1) the study design, (2) dose titration, (3) adequacy of dose achieved, (4) duration of the fixed dose period, (5) the number and wide geographical location of study sites needed to conduct the study, (6) the level of refractoriness of the study population, and (7) differential response to study treatments over time in study, particularly the placebo response. A review of randomized, parallel-group, placebo-controlled trials in neuropathic pain found that the magnitude of the placebo response accrues slowly and continually, whereas pain reduction in response to active treatments manifests more rapidly, before leveling off [21]. Such a pattern of placebo response occurred over the course of the present study and possibly explains why pregabalin demonstrated a significant treatment effect on mean pain intensity in earlier weeks, but not in the primary analysis at week 15. Moreover, the efficacy of pregabalin observed in the earlier weeks of this trial is consistent with the results from a prior, shorter trial with an 8-week double-blind period [10]. Although subjects in this study by Van Seventer et al. had an equally diverse set of etiologic mechanisms associated with chronic neuropathic pain [10], it should also be noted that the duration of the syndromes was nearly 50% shorter (4.4 vs 8 years in this study) and, therefore, the studies may not be strictly comparable.

In future studies designed for a post-traumatic neuropathic pain study population, a few considerations are worth noting. For example, a retrospective analysis demonstrated the effect size was larger in the post-surgical vs non-surgical subgroup (− 0.43 vs 0.05, respectively), and nominal unadjusted *P* value was 0.04 compared with placebo (data on file). However, this is caveated in that the surgical and non-surgical subgroup analyses were not preplanned prior to unblinding, were conducted post hoc and not adjusted for multiple comparisons. It may be, for example, that post-surgical traumatic pain contrasted with non-surgical pain is diagnosed with greater definitiveness and/or that such subjects can rate their pain and change in pain better than non-surgical subjects. Thus, it would be premature to suggest meaningful significance to this finding; however, this finding could be considered or explored in future research. Fixed-dose studies in contrast to flexible dosing in registration studies might help to address any potential concerns for the adequacy of dose studied. When feasible, using countries with a well-established research infrastructure for neuropathic pain research may be advisable. Performing the final assessment 1 week prior to the end of the study while not making this explicitly apparent to the subjects may potentially avoid the presumed anticipatory narrowing of the treatment difference observed at the end of the current study.

The tolerability of pregabalin observed in this study was consistent with the known profile of pregabalin in the management of NeP [22, 23]. Adverse events were preponderantly dizziness, somnolence, and fatigue, which generally resolved as treatment continued. Treatment-emergent AEs of all-causality resulted in permanent withdrawal from the study for 13 patients in the pregabalin group and 15 in the placebo group. The two SAEs that occurred among pregabalin-treated patients were not ascribed to the study drug.

A few limitations warrant consideration. The methodology included the most recent recommendations intended to enhance assay sensitivity; some of these have face validity but have yet to be empirically tested [12, 24]. A related limitation was the lack of capacity to determine the effect of the recommended methods on assay sensitivity. Many variables related to study design influence assay sensitivity making it difficult to draw conclusions from a single study. The acute mechanisms of nerve injury within the enrolled population were etiologically diverse, and it may be difficult to discern in some cases, whether a peripheral nerve injury and corresponding deficits characterized prior to surgery were not worsened by tissue manipulation, electrocautery, and/or incisions of the required surgery. Eighteen percent of participants did not complete the study, and the corresponding loss of data may have affected the primary result. Treatment-emergent side effects of pregabalin had the potential for partial unblinding, which may have led to bias. Finally, performing multiple analyses and basing conclusions of statistical significance on *p* values < 0.05 may lead to false-positive inferences; however, considering that the preponderance of clinically relevant secondary analyses yielded *p* values < 0.05, the totality of the data appears to support a clinical benefit for pregabalin in PTNP.

The primary analysis of this trial was not statistically significant; however, secondary analyses of multiple outcome measures were. Statistically significant changes were relatively modest, and there is no consensus on the magnitude of a group difference for the 0–10 NRS that is considered clinically meaningful. The efficacy of pregabalin in PTNP merits further study in light of promising findings in clinically relevant outcomes, the low rate of SAEs, the positive results of a previous trial, established efficacy in other chronic NeP syndromes, and the lack of evidence-based treatments for PTNP.

## Key points

*Question* Is pregabalin efficacious and tolerable for the treatment of chronic, post-traumatic neuropathic pain?

*Findings* In a double-blind, randomized international study of 542 evaluable patients (pregabalin *n* = 274) of whom approximately half had post-surgical neuropathic pain, the primary efficacy analysis did not demonstrate a statistically significant difference between active treatment and placebo in change from baseline to week 15 (*p* = 0.1823). However, comparisons for key secondary outcome measures yielded *p* values < 0.05 favoring pregabalin. Safety and tolerability were consistent with the known profile of pregabalin.

*Meaning* Additional studies are needed to characterize the efficacy and tolerability of pregabalin for chronic, post-traumatic neuropathic pain.

## Electronic supplementary material

Below is the link to the electronic supplementary material.

Supplementary material 1 (DOCX 104 KB)

Supplementary material 2 (DOCX 127 KB)
