# Supplementary material for: Efficacy of pregabalin in post-traumatic peripheral neuropathic pain: a randomized, double-blind, placebo-controlled phase 3 trial
Source: J Neurol. 2018 Sep 21;265(12):2815–24. doi: 10.1007/s00415-018-9063-9 (PMC6244661; doi:10.1007/s00415-018-9063-9)
Supplement: Supplementary file 1 — Supplementary material 1 (DOCX 104 KB) [file 415_2018_9063_MOESM1_ESM.docx]

**
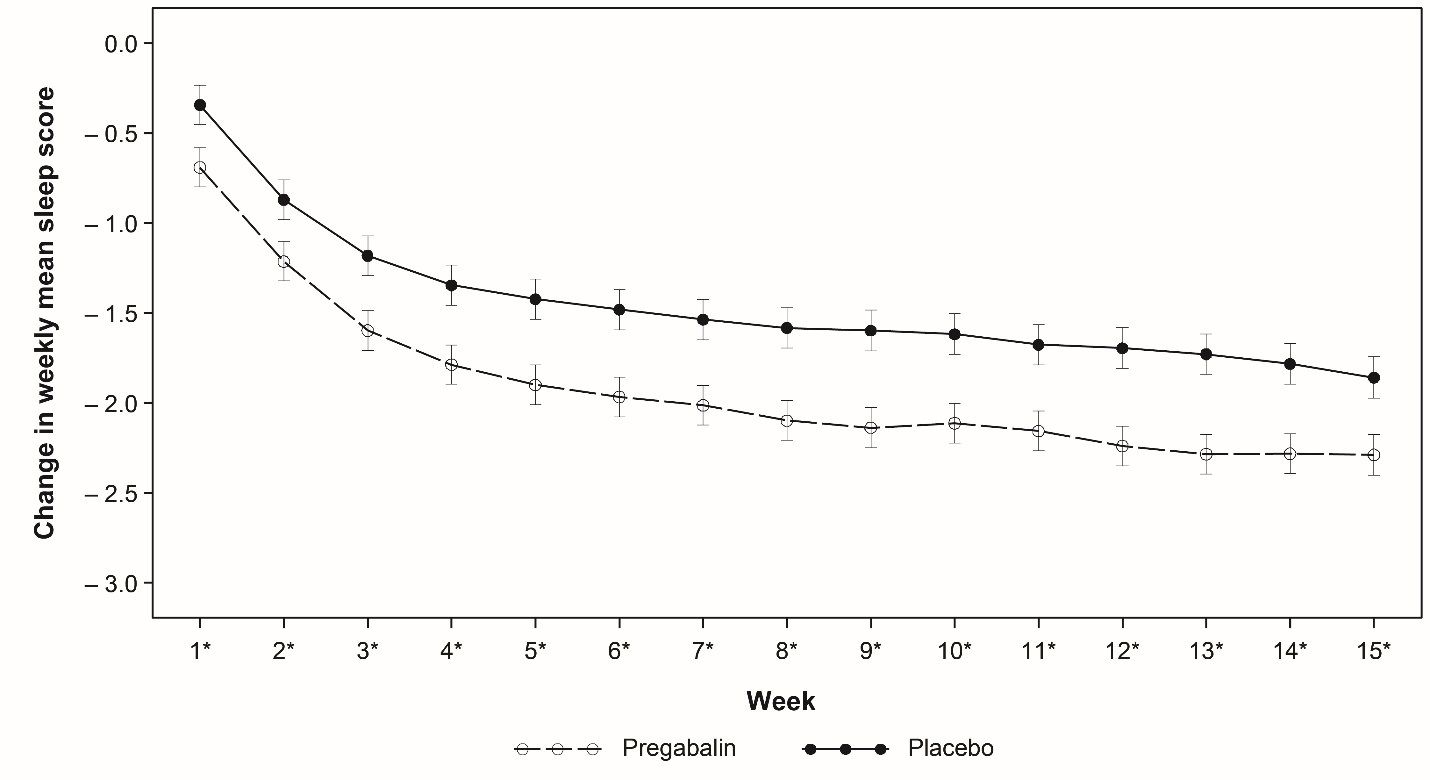
**

**Fig. S2** Change from baseline in weekly mean sleep interference rating scale score (daily sleep diary) – ITT population. *Unadjusted *p* < 0.05 from MMRM analysis.

*Unadjusted p < 0.05 from MMRM analysis. Changes in weekly mean sleep score ± standard error were estimated from mixed-model repeated measures (MMRM) model. Weekly mean sleep scores are derived from the daily sleep numerical rating scale (NRS) and calculated as the mean of the available scores in the 7 days. Generally, week “n” mean sleep score is defined as the mean of the 7 daily sleep diary ratings from day 2+7*(n-1) to day 1+7*n. At least four entries within the last 7 days are required to calculate a mean score. NRS ranged from 0 (“pain does not interfere with sleep”) to 10 (“pain completely interferes with sleep” [unable to sleep due to pain]), with higher scores indicating increased sleep interference. ITT intention to treat.
