# Supplementary figures and images for: Efficacy of pregabalin in post-traumatic peripheral neuropathic pain: a randomized, double-blind, placebo-controlled phase 3 trial
Source: J Neurol. 2018 Sep 21;265(12):2815–24. doi: 10.1007/s00415-018-9063-9 (PMC6244661; doi:10.1007/s00415-018-9063-9)

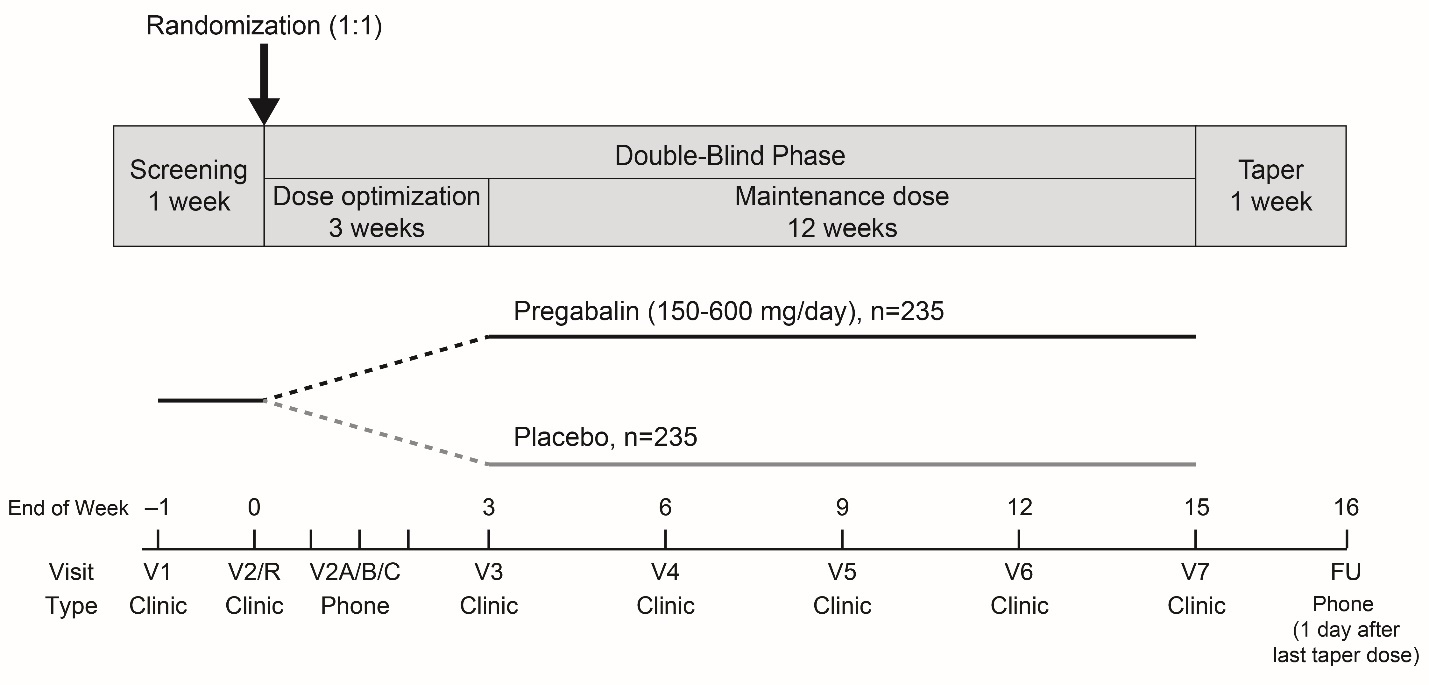


**Fig. S1** Study design

Supplement: Supplementary file 2 — Supplementary material 2 (DOCX 127 KB) [file 415_2018_9063_MOESM2_ESM.docx]
